# Environmental dynamics of *Campylobacter jejuni* genotypes circulating in Luxembourg: what is the role of wild birds?

**DOI:** 10.1099/mgen.0.001031

**Published:** 2023-06-05

**Authors:** Louise Hock, Malte Herold, Cécile Walczak, Alexandra Schoos, Christian Penny, Henry-Michel Cauchie, Catherine Ragimbeau

**Affiliations:** ^1^​ Luxembourg Institute of Science and Technology (LIST), Environmental Research and Innovation (ERIN) Department, 41 rue du Brill, L-4422 Belvaux, Luxembourg; ^2^​ Laboratoire National de Santé (LNS), Epidemiology and Microbial Genomics, 1 rue Louis Rech, L-3555 Dudelange, Luxembourg; ^3^​ Laboratoire de Médecine Vétérinaire de l’Etat (LMVE), 1 rue Louis Rech, L-3555 Dudelange, Luxembourg

**Keywords:** *Campylobacter *, genotyping, wild birds, surface water, diffuse outbreak

## Abstract

*
Campylobacter jejuni* is the leading cause of bacterial gastroenteritis worldwide, but, unlike other foodborne pathogens, is not commonly reported as causing outbreaks. The population structure of the species is characterized by a high degree of genetic diversity, but the presence of stable clonally derived genotypes persisting in space and time, and potentially leading to diffuse outbreaks, has recently been identified. The spread of these recurring genotypes could be enhanced by wild birds, suspected to act as vectors for a wide range of microorganisms that can be transmissible to other animals or humans. This study assessed the genetic diversity of *C. jejuni* carriage in wild birds and surface waters to explore a potential link between these environments and the persistence over years of recurring lineages infecting humans in Luxembourg. These lineages corresponded to over 40 % of clinical isolates over a 4 year period from 2018 to 2021. While mainly exotic genotypes were recovered from environmental samples, 4 % of *C. jejuni* from wild birds corresponded to human recurring genotypes. Among them, a human clinical endemic lineage, occurring for over a decade in Luxembourg, was detected in one bird species, suggesting a possible contribution to the persistence of this clone and its multi-host feature. Whereas 27 % of wild birds were carriers of *C. jejuni,* confirming their role as spreader or reservoir, only three out of 59 genotypes overlapped with recurring human strains. While direct transmission of *C. jejuni* infection through wild birds remains questionable, they may play a key role in the environmental spreading of stable clones to livestock, and this issue merits further investigation.

## Data Summary

A collection of *C. jejuni* isolates originating from wild bird faeces and surface waters sampled between 2019 and 2021 was built. The genome accession numbers and associated metadata can be found in Table S1, available in the online version of this article. Sequenced raw reads have been uploaded to ENA and are available under the accession project number PRJEB57730. The genome accession number and associated metadata can be found in Table S2. Sequenced raw reads of recurring genotypes isolated from human, animal and food samples are available under the accession project numbers PRJEB40465 and PRJEB60495. The authors confirm all supporting data, code and protocols have been provided within the article or through supplementary data files.

**Impact Statement**

As the leading cause of bacterial gastroenteritis worldwide, epidemiological and source attribution studies of *Campylobacter* are essential to manage health risks and guide public health policies in the control of foodborne illnesses. In addition, the hypothesis of *Campylobacter* diffuse outbreaks has recently emerged due to the persistence of some genotypes over space and time. Here, we investigated the role played by wild birds and surface waters in the cycle of transmission of *C. jejuni *’s recurring genotypes in Luxembourg. Wild birds are known to play a significant role in the contamination of surface waters but the link to human illnesses remained unclear. We identified *C. jejuni* in more than a quarter of wild bird samples, confirming their role as an environmental reservoir. We also detected some human recurring genotypes in wild birds that were also isolated in livestock over the same period. Consequently, the results suggested that wild birds could contribute to the field–farm–fork continuum of the recurring stable clones of *C. jejuni *. We provided new insight into the potential role of the environment, and more specifically wild birds, in the persistence and spread of certain stable lineages causing campylobacteriosis.

## Introduction

Since 2005, *Campylobacter* species have been the main zoonotic agents causing bacterial diarrhoeal diseases in the European Union (EU) and campylobacteriosis ranks third in death due to foodborne infection, behind listeriosis and salmonellosis [1]. In 2020, the reported EU-wide incidence of campylobacteriosis was 40.3 cases per 100 000 population, with one of the highest rates of 116.4 recorded in Luxembourg [1]. Beyond gastroenteritis, *Campylobacter* infection may occasionally trigger the development of sequelae, such as reactive arthritis, Guillain-Barré syndrome and irritable bowel syndrome [2]. In human cases, *Campylobacter jejuni* is predominant (83.1 %), followed by *Campylobacter coli* (10.8%) [3].

Contrary to the epidemic curve commonly detected in cases of foodborne outbreaks, stable genetic lineages of *Campylobacter* emerge as an endemic pattern, causing regular infections in humans. The recurrence of these *C. jejuni* genetic variants potentially suggests their ability to persist over space and time, in environments and subsequently along the field–farm–fork continuum [4]. These characteristics confer the potential for geographical spread causing diffuse outbreaks.

Poultry is considered the primary source or reservoir of *Campylobacter* human infections [5]. Livestock such as pigs, bovines and sheep, domestic animals such as dogs and cats, but also wild animals such as birds, penguins, reptiles, turtles, bats, kangaroos and houseflies are considered secondary reservoirs. *Campylobacter* is mainly transmitted to humans by ingestion of contaminated food and direct contact with carrier animals [5]. For instance, undercooked chicken meat, raw milk consumption and cross-contamination during the handling of raw meat are the main causes of campylobacteriosis in humans [5]. Nevertheless, the environment, wildlife and insects are also implicated in the transmission of *Campylobacter* to poultry farms [6]. *C. jejuni* is a thermotolerant species with an optimal growth temperature of 42 °C, which corresponds to the body temperature of birds [7]. This feature confirms its ecotype and consequently its asymptomatic carriage into the digestive tract of wild and domestic birds [7]. Wild birds, including migratory species, can become vectors for a wide range of microorganisms, including *Campylobacter *, that can be transmissible to other animals or humans [8–10]. Moreover, some wild bird species are adapted to anthropogenic environments and routinely come into close contact with livestock, domestic animals and people, facilitating microorganism transfers. These transfers mainly occur orally through the ingestion of food and water contaminated by bird faeces and, to a lesser extent, via direct contact with or inhalation from contaminated air conditioners or vents [11]. In addition, surface waters represent a sink collecting bacteria from different animal reservoirs [12]. Several previous studies have shown the major role played by wildlife, and especially wild birds, in *C. jejuni* contamination of surface waters, spreading the bacteria to the environment [12–16].

This study aims to better understand the role of our environment in the cycle of *Campylobacter* transmission by exploring the dynamics involving wildlife such as birds. As far as we know, this is the first study on the prevalence of *Campylobacter* in wild birds in Luxembourg. With a focus on recurring and epidemic genotypes in human infections, we seek to investigate their role as potential reservoirs and spread vectors. The prevalence of *C. jejuni* in a diverse collection of wild birds was assessed and their contribution in the transmission of campylobacteriosis to humans was determined by sequencing and genotype comparison. Ultimately, the identification of key players and the transmission pathways of recurring genotypes will improve preventive measures against human outbreaks of *Campylobacter *.

## Methods

### Collection of human isolates

Between 2018 and 2021, the Laboratoire National de Santé (LNS), the public health laboratory in Luxembourg, received around 73 % of the notified *C. jejuni* isolates from patient cases. A total of 1131 isolates were processed by next-generation sequencing (NGS).

### Collection of veterinary and food isolates

This collection was carried out via a framework of food official controls at the national level and the EU-coordinated zoonoses monitoring programme, all conducted in Luxembourg by the state laboratory of veterinary medicine. A total of 240 *C *. *jejuni* isolates were transferred to LNS for sequencing and included the following sources: poultry farms (*n*=7), bovine faeces (*n*=133), chicken (*n*=83), turkey (*n*=16), and food with species not defined (*n*=1). All isolations were performed between 2018 and 2021.

### Collection of surface water and wild bird isolates

Between February 2019 and June 2021, 88 surface water samples were collected from Luxembourg rivers [Alzette, Moselle (including Remerschen ponds) and Sûre] and lakes (Upper-Sûre reservoir, Weiswampach). During the same period, 362 faecal samples from 42 wild bird species were collected in collaboration with the Luxembourg bird protection association Natur and Emwelt. Fresh faecal samples were collected in 10 ml of sterile PBS and stored on ice for less than 6 h before filtration.

### *Campylobacter* isolation by a passive filtration method (PFM)

Bacteria were concentrated from water samples by membrane filtration through a 0.45 or 0.65 µm sterile filter membrane (Sartorius cellulose nitrate membrane filter, diameter 47 mm). Depending on the water turbidity, adapted volumes of 100–500 ml were filtered and the filter was placed retentate-up on mCCDA plates (Oxoid; PO5091A). The filter was removed from the agar surface after 18–20 h of incubation at 42 °C in sealed jars (Anaerojar 2.5; Oxoid, AG0025A) under microaerobic conditions (CampyGen gas-generating system; Oxoid, CN0025). After 24–72 h of supplementary incubation under microaerobic conditions, *Campylobacter *-like colonies were selected. Between one and five colonies were selected for subtyping steps, but in cases of abundant biomass growth, up to ten colonies were isolated per sample. Strains were isolated by multiple streaking steps on mCCDA and chocolate agar with Vitox (Oxoid, PO5090A). Faecal samples were well vortexed and 400 µl was transferred on a 0.65 µm filter (Sartorius cellulose nitrate membrane filter, diameter 47 mm) placed on an mCCDA plate and incubated as previously described.

*Campylobacter* isolates were identified at the species level using *hipO/glyA* gene PCR amplification [17] or MALDI-TOF MS [18]. All isolates were stored at −80 °C in FBP medium [19].

### Whole-genome sequencing and genotyping of *C. jejuni* isolates

DNA was extracted using the QIAamp DNA Mini Kit (Qiagen, 51306) according to the manufacturer’s instructions. DNA was quantified with the Qubit 2.0 Fluorometer (Invitrogen, Q32866) and the Qubit dsDNA HS Assay kit (Life Technologies, Q32851). DNA concentration was adjusted to be within the range 3–17 ng µl^–1^ for subsequent sequencing. Libraries were prepared using the Nextera DNA Flex Library Prep Kit (Illumina) and sequenced either on the Miseq or the Miniseq platforms (Illumina) achieving 250 and 150 bp paired-end reads, respectively. For human, food and animal isolates, *de novo* assemblies were processed by using Velvet version 1.1.04 in 2018 and SKESA version 2.3.0 from 2019 to 2021 implemented on SeqSphere+ v8.3.1 (Ridom) [20]. For environmental and wildlife isolates, the paired-end raw read data were *de novo* assembled using SPAdes version 3.11.1 (default parameters) [21] implemented on SeqSphere+ v8.3.1. Multi-locus sequence typing (MLST) (*n*=7 loci) combined with *porA* and *gyrA* loci (PubMLST database [22, 23]) and SeqSphere+ core genome MLST (cgMLST) (*n*=637 loci) were determined by a scheme developed by Ridom SeqSphere+, and *Campylobacter* isolates were classified by genotypes with the following definition: complex type (CT) assigned by the cgMLST scheme from SeqSphere+ extended MLST type. The SeqSphere+ cgMLST has previously showed a high congruence with other typing schemes [4]. The extended MLST is composed of the sequence type (ST) (classical MLST scheme with seven loci) combined with the *gyrA* and *porA* loci [4].

### Statistical analyses

The diversity of *C. jejuni* was measured by Simpson’s Diversity Index [24]. Measurements of proportion and their confidence interval (95 % CI) followed a binomial law approximated by a normal law. *Campylobacter* prevalence in different groups was analysed by a chi-squared homogeneity statistical test (Rstudio 2022.02.0). Bonferroni correction was applied for pairwise comparison. All *P*-values<0.05 were considered statistically significant. Minimum spanning trees of wild bird and surface water isolates were constructed by PHYLOViZ based on typing of the 637 loci of cgMLST (SeqSphere+) and nine loci of extended MLST (MLST-*gyrA*-*porA*).

## Results

### Genetic diversity of Campylobacter isolates in humans

Between 2018 and 2021, 1712 cases of campylobacteriosis were reported in Luxembourg. In total, 1131 *C *. *jejuni* isolates were sequenced and assigned to 593 genotypes (CT-ST*-porA-gyrA*), corresponding to a Simpson diversity index of 0.995 (95 % CI: 0.994–0.996) and reflecting a highly diverse community (Table 1). A total of 59 recurring genotypes, detected at least three times in human infections within the study period, were identified as regrouping into 464 isolates corresponding to 41 % (95 % CI: 38–44 %) of *Campylobacter* human clinical cases (Table S3).

**Table 1.** Distribution of the human clinical *Campylobacter* notifications in Luxembourg between 2018 and 2021

	No. of cases/isolates				
	2018	2019	2020	2021	Total
No. of national notifications for campylobacteriosis	625	271	227	589	1712
No. of *C. jejuni* isolates sequenced	477	255	208	191	1131
No. of human cases associated with recurring genotypes (%)	219 (45.91 %)	90 (35.29 %)	92 (44.23 %)	63 (32.98 %)	464 (41.03 %)
Confidence interval (95%) of the no. of human cases associated with recurring genotypes	42–51 %	29–41 %	37–51 %	26–40 %	36–44 %

**Fig. 1.**
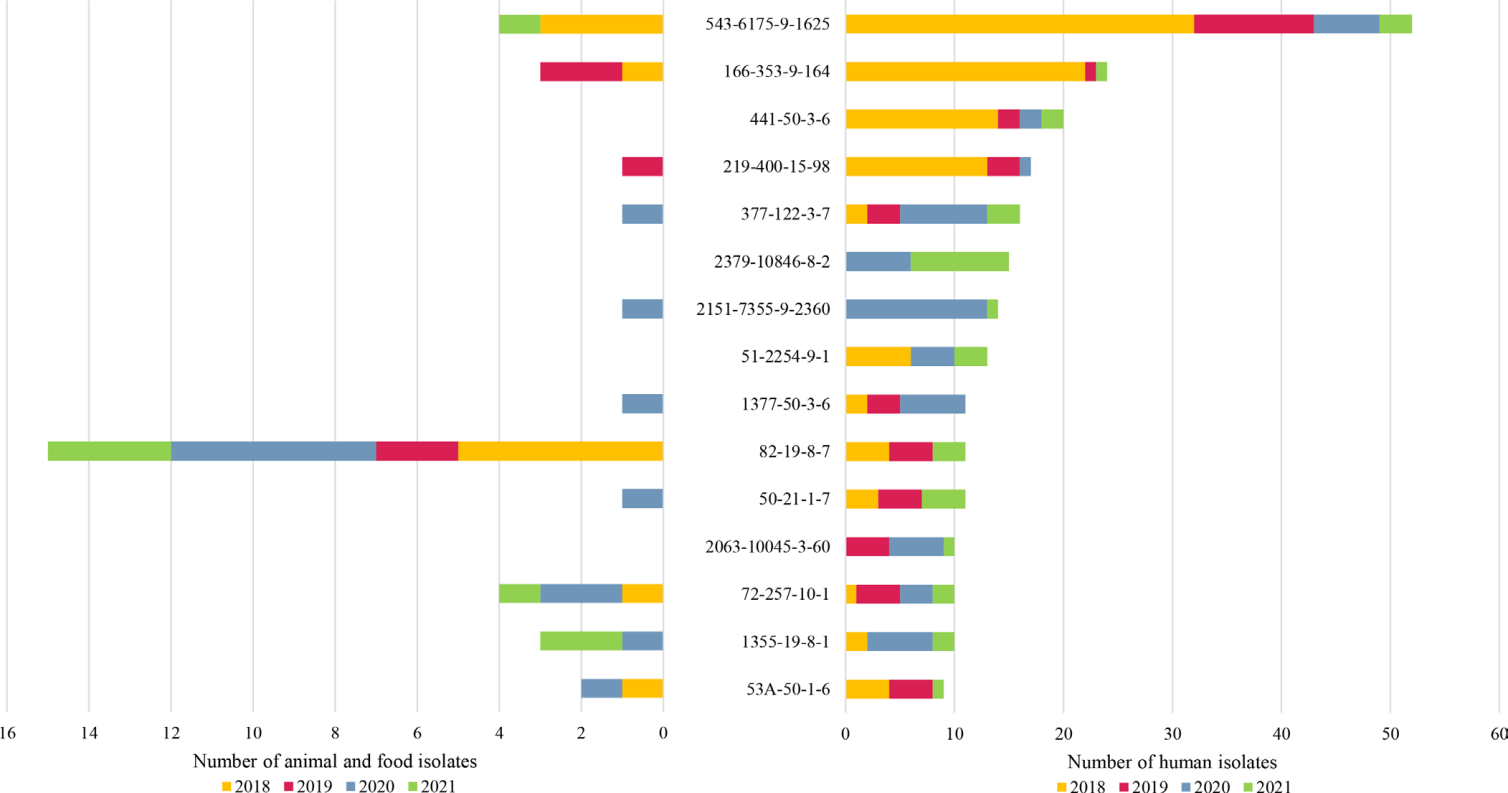
Time repartition of the top 15 *C *. *jejuni* recurring genotypes in human infections between 2018 and 2021 and their corresponding time repartition in food and animal samples.

Genotype CT543-ST6175*-gyrA*9*-porA*1625 (known as recurring lineage D in Luxembourg [4]) was the most recurrent genotype with 32 detections in human cases in 2018, 11 in 2019, six in 2020 and three in 2021 (Fig. 1). The genotypes CT2151-ST7355*-gyrA*9*-porA*2360 and CT2379-ST10846*-gyrA*8*-porA*2 were the most recurrent in 2020 and 2021, respectively.

### Genetic diversity in food and animal isolates

From the 240 isolates of non-human sources, 158 genotypes were identified corresponding to a Simpson diversity index of 0.990 (95 % CI: 0.985–0.995). Approximately 35 % (95 % CI: 29–42 %) of these isolates (*n*=77) shared genotypes classified as recurring genotypes in humans (*n*=27 genotypes) (Table S3). The profiles CT82-ST19-*gyrA*8-*porA*7 (known as endemic lineage A in Luxembourg [4]) and CT46B-ST21-*gyrA*8-*porA*7 were commonly found in bovine faecal samples (*n*=13 and *n*=12, respectively). The genotypes CT543-ST6175-*gyrA*9-*porA*1625 (recurring lineage D in Luxembourg [4]) and CT1868-ST10025-*gyrA*3-*porA*92 were isolated from poultry sources only (farms and food) with *n*=4 and *n*=6, respectively. A total of 11 recurring genotypes classified in the top 15 (Fig. 1) were identified in non-human sources as well.

### Occurrence of *C. jejuni* in the environment: wild birds and surface waters

Of 362 wild bird faeces collected, the presence of *C. jejuni* was confirmed in 97 samples (27%, 95 % CI: 22–31 %) belonging to 20 bird species and 110 strains were isolated. In 89 % (86 out of 97, 95 % CI: 82–95 %) of wild bird samples, only one genotype of *C. jejuni* was isolated. However, in nine (B036, B042, B050, B054, B063, B100, B113, B114, B336) and two (B038, B203) samples, two and three different strains were isolated, respectively. All these samples with several different *C. jejuni* isolates come from passerine birds such as common blackbirds (*Turdus merula*), western jackdaws (*Corvus monedula*), carrion crows (*Corvus corone*) and common swifts (*Apus apus*).

**Fig. 2.**
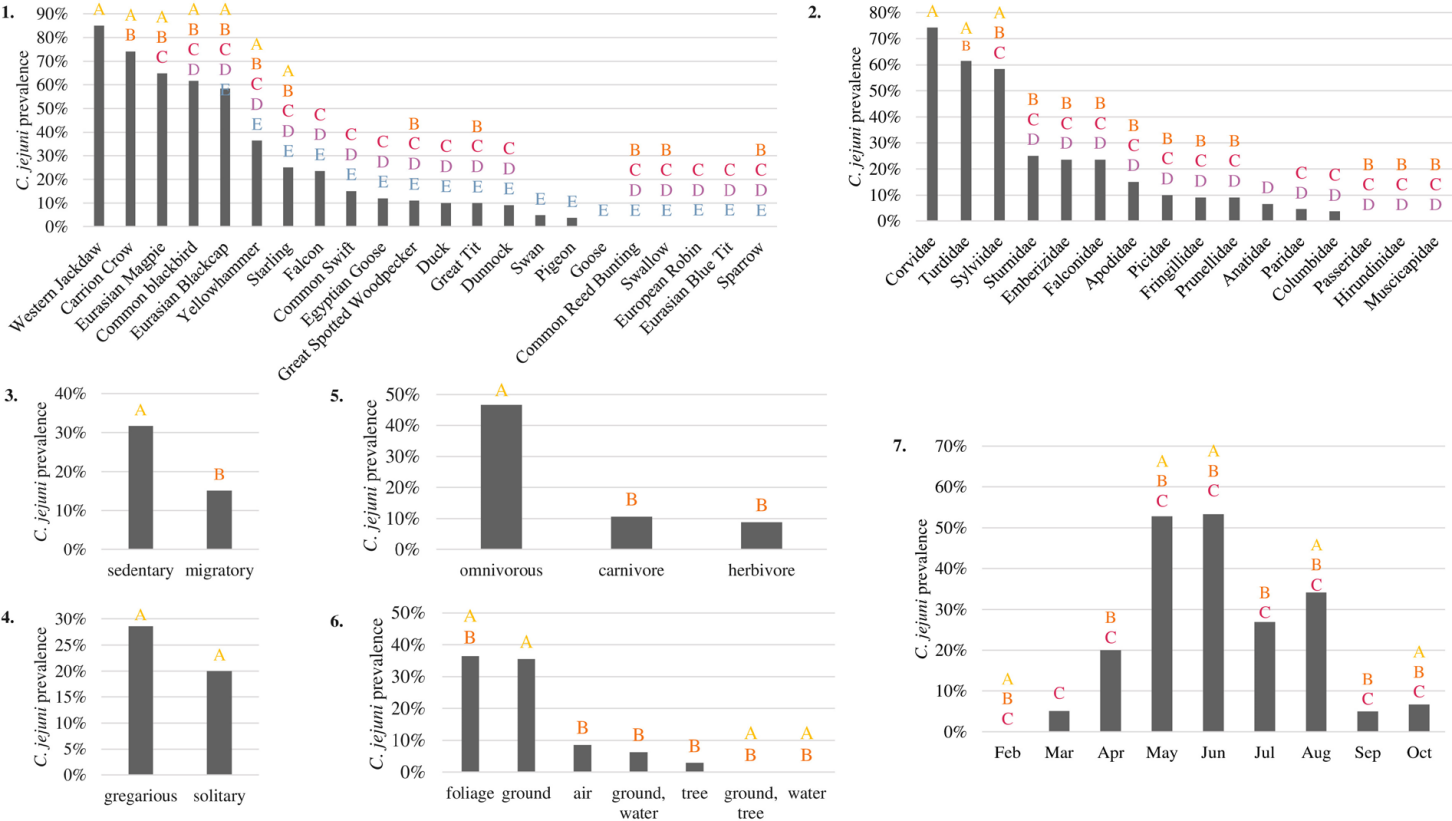
Prevalence of *C. jejuni *-positive samples in wild birds according to different characteristics: (1) bird species, (2) bird family, (3) migratory behaviour, (4) social behaviour, (5) food diet, (6) localization of their food and (7) sampling month. Prevalence is estimated based on at least five samples corresponding to a characteristic. Characteristics sharing the same letter have no statistically different *C. jejuni* prevalence (chi-squared test, Bonferroni correction, *P*=0.05).

Among bird species with at least five samples (*n*=22), the *C. jejuni* prevalence rate ranged from 95 % in western jackdaws (in 19 out of 20 samples) to 4 % among pigeons (*Columba*) (in one out of 27 samples) (Table 2). No *C. jejuni* was isolated from geese (*Anser*, *n*=18), swallows (*Hirundo*, *n*=7), sparrows (*Passer*, *n*=5), common reed buntings (*Emberiza schoeniclus*, *n*=6), European robins (*Erithacus rubecula*, *n*=8) or Eurasian blue tits (*Cyanistes caeruleus*, *n*=12). Corvids [western jackdaws, carrion crows and Eurasian magpies (*Pica pica*); family Corvidae] are the most frequently contaminated bird species followed by blackbirds (family Turdidae) and Eurasian blackcaps (*Sylvia atricapilla*, family Sylviidae) (Fig. 2).

**Table 2.** Prevalence of *C. jejuni* in different bird species Bird characteristics: migration: S, sedentary, M, migratory; social behaviour: S, solitary, Gr, gregarious; diet: C, carnivore, H, herbivore, O, omnivore; feeding: A, air, F, foliage, G, ground, T, tree, W, water. Light grey lines represent species with fewer than five samples.

Order	Family	Common name	Latin name	Migration	Social behaviour	Diet	Feeding	No. of samples	No. of positive samples (%)
Accipitriformes	Accipitridae	Common buzzard	*Buteo buteo*	S	S	C	G	4	1(25)
Anseriformes	Anatidae	Duck	*Anas*	M	Gr	O	G W	10	1 (10)
		Swan	*Cygnus*	M	Gr	O	G W	21	1 (5)
		Goose	*Anser*	M	Gr	H	G	18	0
		Egyptian goose	*Alopochen aegyptiacus*	S	Gr	H	G	25	3 (12)
		Canada goose	*Branta canadensis*	M	Gr	H	G W	1	0
		Snow goose	*Chen caerulescens*	M	Gr	H	G	1	0
Apodiformes	Apodidae	Common swift	*Apus apus*	M	Gr	C	A	20	3 (15)
Columbiformes	Columbidae	Pigeon	*Columba*	S	Gr	H	G	27	1 (4)
Coraciiforms	Alcedinidae	Common kingfisher	*Alcedo athis*	S	S	C	w	1	0
Falconiformes	Falconidae	Falcon	*Falco*	S	S	C	G	17	4 (24)
Passeriformes	Acrocephalus	Reed warbler	*Acreocephalus scirpaceus*	M	S	C	G	1	0
	Aegithalidae	Long-tailed tit	*Aegithalos caudatus*	S	Gr	C	G	1	0
	Carduelinae	Eurasian bullfinch	*Pyrrhula pyrrhula*	S	S	H	T	1	0
	Certhiidae	Short-toed treecreeper	*Certhia brachydactyla*	S	S	C	T	1	0
	Corvidae	Western jackdaw	*Corvus monedula*	S	Gr	O	G	20	17 (85)
		Carrion crow	*Corvus corone*	S	Gr	O	G	27	20 (74)
		Eurasian magpie	*Pica pica*	S	Gr	O	G	17	11 (65)
		Eurasian jay	*Garrulus glandarius*	S	S	O	G	2	1 (50)
	Emberizidae	Yellowhammer	*Emberiza citrinella*	M	Gr	H	G	11	4 (36)
		Common reed bunting	*Emberiza schoeniclus*	S	Gr	O	T	6	0
	Fringillidae	Common chaffinch	*Fringilla coelebs*	S	Gr	O	G	4	1 (25)
		European greenfinch	*Chloris chloris*	S	Gr	H	G	4	0
		Hawfinch	*Coccothraustes coccothraustes*	s	S	H	T	2	0
		Linnet	*Linaria*	S	S	H	G	1	0
	Hirundinidae	Swallow	*Hirundo*	M	Gr	C	A	7	0
	Motacillidae	White wagtail	*Motacilla alba*	M	Gr	C	T	1	0
	Muscicapidae	European robin	*Erithacus rubecula*	S	S	C	A	8	0
	Paridae	Great tit	*Parus major*	S	Gr	C	F	10	1 (10)
		Eurasian blue tit	*Cyanistes caeruleus*	S	Gr	C	T	12	0
	Passeridae	Sparrow	*Passer*	S	Gr	O	G	5	0
	Phylloscopidae	Common chiffchaff	*Phylloscopus collybita*	M	S	C	G T	2	0
	Prunellidae	Dunnock	*Prunella modularis*	S	S	C	G	11	1 (9)
	Remizidae	Eurasian penduline tit	*Remix pendulinis*	M	Gr	C	T	1	0
	Sittidae	Nuthatch	*Sitta*	S	S	C	T	2	0
	Sturnidae	Starling	*Sturnus*	S	Gr	O	G	8	2(25)
	Sylviidae	Eurasian blackcap	*Sylvia atricapilla*	M	S	O	F	12	7 (58)
	Turdidae	Common blackbird	*Turdus merula*	S	Gr	O	G	26	16 (62)
Pelecaniformes	Ardeidae	Grey heron	*Ardea cinerea*	S	Gr	C	w	1	0
Piciformes	Picidae	Great spotted woodpecker	*Dendrocopos major*	S	S	O	T	9	1 (11)
		European green woodpecker	*Picus viridis*	S	S	C	G	1	0
Strigiformes	Strigidae	Long-eared owl	*Asio otus*	S	Gr	C	G	3	1 (33)
						Total: 95 % CI		362	97 (27) 22–31

Migratory behaviour, as well as the diet of birds, are factors impacting their contamination by *C. jejuni *, whereas their social behaviour has no significant influence (Fig. 2). The prevalence of *C. jejuni* is significantly higher in sedentary birds (33 %, 95 % CI: 27–39 %) than migratory birds (15 %, 95 % CI: 8–22 %), and in omnivorous birds (49 %, 95 % CI: 42–57 %) than in carnivorous birds (11 %, 95 % CI: 5–16 %) or herbivorous birds (9 %, 95 % CI: 3–15 %). Furthermore, birds eating by forage on the ground (37 %, 95 % CI: 35–43 %) and foliage (36 %, 95 % CI: 16–56 %) present a significantly higher *C. jejuni* prevalence than those that find their food by hunting in the air, in water or on tree trunks. The prevalence of *C. jejuni* in birds shows a seasonal peak in May and June, with more than 50 % (95 % CI: 45–65 %) of bird isolates infected by *C. jejuni* (Fig. 2g).

In addition, *C. jejuni* was isolated from 11 % (10 out of 88, 95 % CI: 5–18 %) of surface water samples during the same period, between February 2019 and June 2021. The *Campylobacter* species found in surface waters was mainly *C. coli* (* C. coli* was isolated in 66 % of water samples, 58 positive samples, 95 % CI: 56–76 %).

### Genotype distribution of *C. jejun* i in wild birds

Overall, the 110 *C *. *jejuni* isolated from birds were assigned to 80 genotypes, corresponding to a Simpson diversity index of 0.991 (95 % CI: 0.986–0.996), reflecting a diverse population. The most prevalent genotypes were CT251-ST383-*gyrA*1-*porA*73 (5 % of isolates, 95 % CI: 1–10 %), CT2165-ST19-*gyrA*8-*porA*7327, CT414-ST11-*gyrA*7-*porA*217 and CT3165-ST11376-*gyrA*108-*porA*2370 (4 % of isolates, 95 % CI: 1–7 %) (Table S1). The generalist ST45 was the main sequence type isolated (7 %, 95 % CI: 3–13 %) from our collection of wild bird samples. This lineage was overrepresented in a previous study in Finland and its dissemination by migrating birds has already been suggested [10]. Only four isolates from wild birds (4 %, 95 % CI: 1–7 %) corresponded to human recurring genotypes (Table 3). Of these four isolates, two were detected from the same migratory bird species, the common swift (*Apus apus*), but at different isolation times and possessing different genotypes. Genotype CT82-ST19-*gyrA*8-*porA*7, corresponding to the recurring lineage A [4], was found in a great spotted woodpecker sampled in June 2021. Two of three human recurring genotypes found in wild birds were also isolated in bovine, turkey and chicken samples during the same period: CT82-ST19-*gyrA*8-*porA*7 and CT827-ST475-*gyrA*8-*porA*67. The ten *C. jejuni* isolated from surface waters were assigned to nine genotypes, different from those highlighted in wild birds or recurring clinical isolates.

**Table 3.** Human *C. jejuni* recurring genotypes isolated from wild birds

Genotypes	Bird species (no. of isolates)	Sampling period in wild birds	No. of isolates in humans				No. of isolates in animals and food (bovine-turkey- chicken)			
CT-ST-*gyrA-porA*			2018	2019	2020	2021	2018	2019	2020	2021
82-19-8-7	Great spotted woodpecker (1)	June 2021	4	4	0	3	5-0-0	2-0-0	4-1-0	2-0-0
84-1044-27-25	Common swift (1) Eurasian magpie (1)	July 2020 June 2021	2	2	0	1	0-0-0	0-0-0	0-0-0	0-0-0
827-475-5-67	Common swift (1)	July 2019	4	1	0	0	0-0-0	1-0-0	0-1-1	0-0-0

**Fig. 3.**
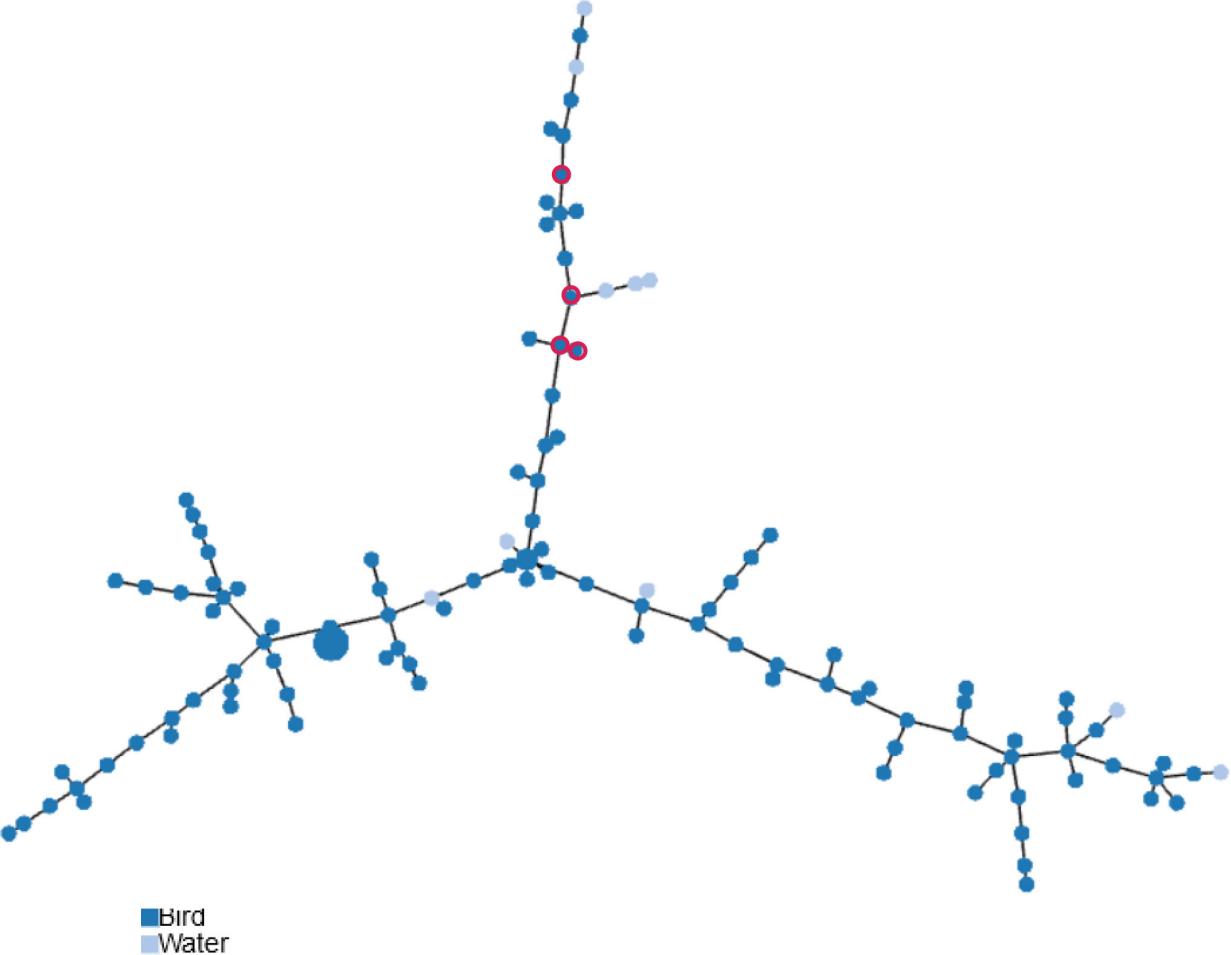
Population structure of C. *jejuni* isolated from wild birds and surface waters based on genotypes. The minimum spanning tree is based on 637 loci of cgMLST (SeqSphere+) and nine loci of extended MLST (MLST-*gyrA*-*porA*). Each circle represents a different genotype profile, where the size of the circle is proportional to the number of isolates and the colour of the circle indicates bird or surface water origin. Human recurring genotypes are circled in red.

The population structure of *C. jejuni* isolated from wild birds and surface waters was visualized using a minimum spanning tree based on genotypes (Fig. 3). Whereas some genotypes from water are regularly linked to those of bird isolates, human recurring genotypes shared by birds correspond to a limited section of the tree.

## Discussion

*C. jejuni* is the leading cause of bacterial diarrhoeal diseases worldwide but seems not to be involved in large outbreaks, in contrast to other foodborne pathogens [1]. The population structure appears to be composed of: (i) a background population with a high genetic diversity, rendering the detection of human clusters difficult, and (ii) the presence of recurrent genotypes, which could persist and spread from a vector/reservoir to another in the environment and provoke clinical cases at an observable frequency [4]. In Luxembourg, between 2018 and 2021, 41 % (95 % CI: 38–44 %) of human campylobacteriosis cases were attributed to 59 *C *. *jejuni* recurring genotypes.

Concerning human epidemiological surveillance, the decrease in the number of isolates reported in 2019 is related to the implementation of culture-independent diagnostic tests (CIDTs) in medical labs. Since 2020, campylobacteriosis has been classified as a notifiable disease and the notification system was changed to include these PCR-confirmed cases whereas, until 2019, isolates from a culture were required to notify the case. CIDTs are easy to perform and give quick results, representing a critical improvement for clinical decision-making. However, by implementing these methods, the collection of isolates is no longer performed and has had a negative impact on public health surveillance in the detection of human clusters and the identification of potential sources [25].

Although poultry is accepted as the main reservoir of *Campylobacter* infections in humans, the role of other avian species in the environmental dynamics of the contamination continuum remains unclear [5]. *Campylobacter* has been isolated from various species of wild birds in the world [26] and *C. jejuni* is the most frequently detected thermotolerant species [27–29]. In our study, among the 362 wild bird faeces collected from 42 wild bird species, *C. jejuni* was isolated in 97 samples, representing 20 bird species. This *Campylobacter* prevalence of 27 % (95 % CI: 22–31 %) in wild birds corresponds to the estimation of a recent literature review about enteric pathogens in wild birds [11]. According to Smith *et al*. [11], *Campylobacter* is more prevalent in wild birds than pathogenic *Escherichia coli* or *Salmonella *. Multiple genotypically different *C. jejuni* were previously isolated from black-headed gulls [30]. Here, some passerine birds (blackbirds, western jackdaws and carrion crows) were contaminated by two or three different genotypes. Corvids (family Corvidae) were the most frequently contaminated bird family, with a contamination rate of 74 % (95 % IC: 64–85 %), in accordance with previous observations of a significanty higher prevalence of *Campylobacter* in Corvidae than n other bird families: 38 % in China [31] and 72 % in Italy [27]. These bird species are abundant across human-inhabited landscapes and could potentially be a vector for food, animal and human contamination [32–34]. Contrary to previous studies regarding pigeon carriage of pathogenic bacteria, including *Campylobacter* found in 26–30 % of samples [28, 35], only 4 % of pigeons collected in our study were contaminated (however, the number of samples is considerably lower in our study). Omnivorous birds, particularly birds that feed by foraging on the ground and on foliage, were more highly contaminated. Previous studies have also shown that carnivorous birds or wild birds that forage on the ground near animal farms are more highly contaminated, because of the faecal–oral transmission from animals to wild birds [26–28, 32]. *C. jejuni* carriage was also linked to seasons with a higher observed prevalence rate during May and June, in accordance with previous observations showing that the spring season brings higher *C. jejuni* rates in birds [29].

Despite their high mobility, migrating wild birds do not appear to be the main carrier of *Campylobacter* leading to diffuse outbreaks because they present a lower contamination rate than sedentary birds. Another study, conducted in Switzerland, also concluded that *Campylobacter* strains isolated from migratory birds were not related to human strains [30]. *C. jejuni* that we had isolated from birds presented a high genomic diversity, but only 4 % (95 % CI: 1–7 %) of isolates corresponded to human recurrent genotypes. Among them, half come from common swifts which are migratory bird species, varying their location between winter and summer. In addition, common swifts mainly eat insects when they are flying [36]. Consequently, insects could be involved in the cycle of transmission of *Campylobacter* to wild birds, similar to the well-known pathway of contamination in poultry production [37–39]. This transmission route involving insects is greatest during summer. As our two human recurring genotypes were isolated from common swifts in July, we could hypothesize that a contamination link between human, insects and birds exists. A similar *Campylobacter* infection rate was reported in the UK where wild birds account for 2.1–3.5 % of human campylobacteriosis cases each year [40]. Consequently, wild birds appear to be a source of a relatively low proportion of human infection by recurring genotypes. Nevertheless, one isolate from birds corresponds to an endemic lineage that has been occurring in human infections for the past 15 years in Luxembourg and more generally in Europe [4]. This lineage is characterized by acclimation to aerobic conditions [41]. Even though *Campylobacter* is generally believed to survive poorly outside the animal host [42], this acclimatation capacity to aerobic environments might improve the environmental survival of this lineage. Its presence in wild birds could be an additional factor contributing to its spatio-temporal persistence [10].

The genetic diversity of *C. jejuni *-infected wild birds is high: the same bird species could carry a variety of *C. jejuni* genotypes. Certain STs have already been observed in wild birds. ST448, found in five of our isolates (carrion crow, Eurasian magpie, starling and western jackdaw), ST951 and ST1540 isolated from carrion crow and western jackdaw respectively have also been isolated in daurian jackdaw in China and in various wild birds in the UK, Japan and USA [31]. ST267 (isolated from carrion crow, common blackbird and chaffinch) has been found among blackbirds in Sweden and various bird species in Italy, and ST48 (isolated from carrion crow) and ST1044 (isolated from common swift and Eurasian magpie) were also shared in human cases and birds in Italy [31, 43–45]. These STs were also found in human infections. The generalist ST45 was the main isolate (7%) from our wild bird sample collection and was also previously isolated from 16 % of wild birds in Italy [46]. This lineage was also overrepresented in a previous study in Finland and its dissemination by migrating birds has already been suggested [10]. Our study confirmed the association of this genotype with the wild bird reservoir. Most STs were shared among different bird species, but certain ones were shown to be species-specific: ST6313, ST10813 and ST11376 were found only in Eurasian blackcap, western jackdaw and yellowhammer, respectively. This bird species specificity of some genotypes has already been described by Marotta *et al*. for isolates from Italy [46].

In surface waters, unlike wild birds, *C. coli* is the main *Campylobacter* species that we have isolated. Moreover, *C. jejuni* isolated from surface waters corresponded to genotypes different from those of wild birds or recurring clinical isolates. Surface water contamination appears not to be associated with human recurring or wild bird isolates [47]. However, the frequent presence of *C. coli* could disrupt the isolation of *C. jejuni* strains because we selected a limited number of colonies. In addition, whereas certain strains may survive in water for up to several months, some of them could be in a viable but not cultivable state even if they are still infectious [48]. Consequently, our knowledge regarding *C. jejuni* contamination of surface waters and diversity may be biased.

**Fig. 4.**
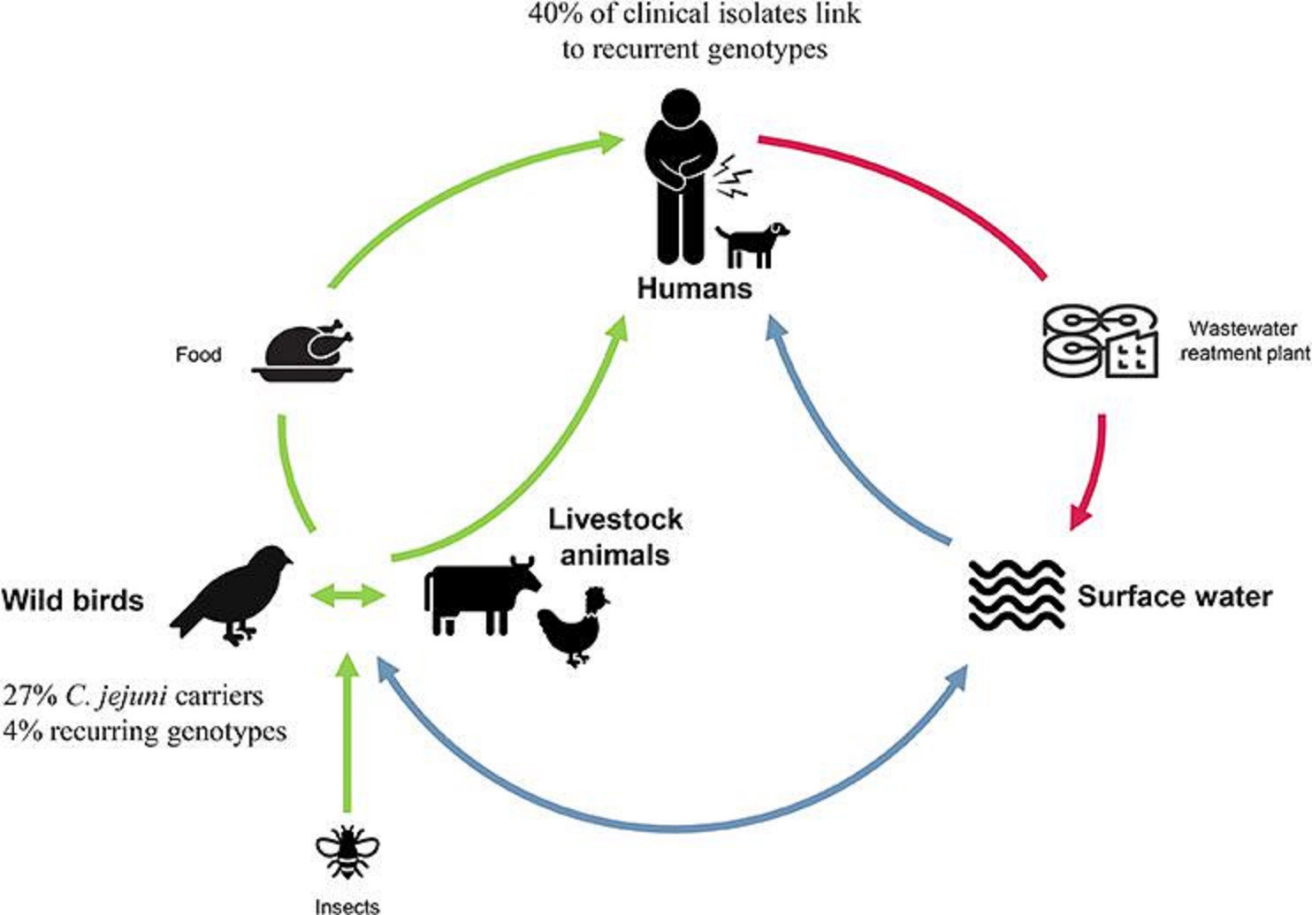
Hypothetical *C. jejuni* contamination cycle. Used icons come from Microsoft, thenounproject.com and shutterstock.com.

The genetic relatedness between bacteria of wild birds, livestock and human origin suggests that crossover is rare and, in fact, most of the strains are host-adapted [11]. Whereas a large proportion of human recurring genotypes could not be attributed to a specific source, several persistent lineages were identified in wild birds, bovines, chickens and turkeys. These findings were confirmed by uploading the genomes of these related strains in the publicly available Bacterial Isolate Genome Sequence database (BIGSdb) [49]. The concordance of the typing systems in classifying these strains corroborated the spread of these lineages in these different hosts and in humans (Table S4). Several genotypes, potentially with specific metaphenotypes [41], could be multi-host and present in different environments/reservoirs/vectors (Fig. 4). In the contamination cycle, surface waters played the role of a sink, collecting strains from multiple hosts following various connections: for example human strains via wastewater, poultry strains via agricultural effluents and wild bird strains via animal faeces [12]. *C. jejuni* contaminating humans could be disseminated in surface waters via wastewater. By contact with these surface waters, animals such as bovines, chickens and wild birds could also become contaminated. Via their faeces, these animals contaminated fields, which become a vector of contamination for other animal food production and waters [47, 50]. Moreover, indirect exposure to surfaces contaminated with bird faeces has been linked to disease in young children [51].

This study had some limitations. Even if we were mindful to sample only birds that had recently arrived and had no antibiotic treatment, the wild bird faeces sampling could be biased by sampling of several wild birds in a wildlife rescue centre. Moreover, despite the diversity of species sampled, some bird species were not captured. In addition, the *Campylobacter* diversity recovered from bird and water samples relies on the isolation media used, the cultivable form of the bacteria, the competitive microflora present in the samples and the number of strains examined for sequencing. One way to overcome these drawbacks could be the development of culture-independent tools, such as the strain-typing metagenomic method [52].

To summarize, birds are asymptomatic carriers and could be a vector of *Campylobacter* infection: more than a quarter of birds are contaminated by *Campylobacter *. Whereas the transmission routes of human recurring *Campylobacter* by wild birds or surface waters appear to be proportionally low, wild birds could contribute to environmental contamination and, consequently, to the potential spread of these recurring genotypes. The *Campylobacter* population could generally be structured into different ecotypes: reservoirs, responding to human infection, corresponding to different genotypes. Our research has illustrated that some persistent genotypes are multi-host, overlapping human, wild birds and livestock. As previously noted, these recurring genotypes have higher adhesion/biofilm formation capacity than sporadic genotypes, contributing to the survival and spread of these stable genomic lineage [41]. The monitoring of *C. jejuni* genotypes in different environments and a better understanding of the contamination cycle can not only shed light on the epidemiology of campylobacteriosis in Luxembourg but also on diffuse outbreaks of this high-priority foodborne pathogen around the world.

## Supplementary Data

Supplementary material 1
