# Supplementary material for: Environmental dynamics of Campylobacter jejuni genotypes circulating in Luxembourg: what is the role of wild birds?
Source: Microb Genom. 2023 Jun 5;9(6):mgen001031. doi: 10.1099/mgen.0.001031 (PMC10327502; doi:10.1099/mgen.0.001031)
Supplement: Supplementary material 1 [file mgen-9-1031-s001.pdf]

Supplementary Table 1 – Metadata of bird and water *C. jejuni* isolates: PubMLST and ENA idea, strain ID, Sampling date and sample ID, Sampling type (bird or surface water), Birds species or water origin, Sequence Type, Complex Type, allele of *gyrA* and *porA* and genotype.

PubMLST ID	ENA Biosample	Strain ID	Sampling date	Sample ID	Sampling type	Bird species/water origin	ST	Complex Type	fn_gyrA	porA	Genotype
112526	SAMEA112151025	B015-230519-01	23/05/2019	B015	Bird	Swan	45	2052	7	467	2052-45-7-467
112527	SAMEA112151026	B021-070519-01	07/05/2019	B021	Bird	Carrion crow	48	153	3	7	153-48-3-7
112528	SAMEA112151027	B023-070519-01	07/05/2019	B023	Bird	Carrion crow	448	2154	102	2353	2154-448-102-2353
112306	SAMEA112151028	B024-230519-01	23/05/2019	B024	Bird	Common blackbird	1080	2300	74	130	2300-1080-74-130
112529	SAMEA112151029	B025-070519-02	07/05/2019	B025	Bird	Carrion crow	677	942	19	276	942-677-19-276
112530	SAMEA112151030	B026-230519-01	23/05/2019	B026	Bird	Eurasian magpie	448	2154	102	2353	2154-448-102-2353
112531	SAMEA112151031	B027-070519-01	07/05/2019	B027	Bird	Eurasian magpie	45	2155	7	49	2155-45-7-49
112532	SAMEA112151032	B028-230519-01	23/05/2019	B028	Bird	Carrion crow	2491	2156	11	1634	2156-2491-11-1634
112533	SAMEA112151033	B029-110619-02	11/06/2019	B029	Bird	Eurasian magpie	448	2624	31	860	2624-448-31-860
112534	SAMEA112151034	B033-110619-01	11/06/2019	B033	Bird	Starling	448	2624	31	860	2624-448-31-860
112535	SAMEA112151035	B036-110619-01	11/06/2019	B036	Bird	Western jackdaw	1540	2157	40	860	2157-1540-40-860
112537	SAMEA112151036	B036-110619-03	11/06/2019	B036	Bird	Western jackdaw	10813	2157	11	2351	2157-10813-11-2351
112538	SAMEA112151037	B037-110619-01	11/06/2019	B037	Bird	Western jackdaw	45	2158	7	49	2158-45-7-49
112539	SAMEA112151038	B038-110619-01	11/06/2019	B038	Bird	Western jackdaw	10818	2159	104	391	2159-10818-104-391
112540	SAMEA112151039	B038-110619-02	11/06/2019	B038	Bird	Western jackdaw	10811	2160	36	1279	2160-10811-36-1279
112541	SAMEA112151040	B038-110619-03	11/06/2019	B038	Bird	Western jackdaw	10817	2161	103	2354	2161-10817-103-2354
112542	SAMEA112151041	B039-110619-03	11/06/2019	B039	Bird	Western jackdaw	10820	2162	11	1224	2162-10820-11-1224
112543	SAMEA112151042	B040F-110619-01	11/06/2019	B040	Bird	Eurasian magpie	11002	2625	99	2347	2625-11002-99-2347
112544	SAMEA112151043	B041-110619-02	11/06/2019	B041	Bird	Carrion crow	4279	2163	31	1638	2163-4279-31-1638
112545	SAMEA112151044	B042-110619-01	11/06/2019	B042	Bird	Carrion crow	11003	2626	36	127	2626-11003-36-127
112546	SAMEA112151045	B042F-110619-01	11/06/2019	B042	Bird	Carrion crow	1282	2164	11	2350	2164-1282-11-2350
112547	SAMEA112151046	B045-180619-01	18/06/2019	B045	Bird	Western jackdaw	464	596	3	2348	596-464-3-2348
112548	SAMEA112151047	B046-180619-01	18/06/2019	B046	Bird	Common blackbird	677	38	19	276	38-677-19-276
112549	SAMEA112151048	B047-180619-01	18/06/2019	B047	Bird	Falcon	19	2165	8	327	2165-19-8-327
112550	SAMEA112151049	B050-180619-01	18/06/2019	B050	Bird	Western jackdaw	10813	2166	11	2351	2166-10813-11-2351
112551	SAMEA112151050	B050-180619-02	18/06/2019	B050	Bird	Western jackdaw	1080	2301	74	130	2301-1080-74-130

112552	SAMEA112151051	B051-180619-02	18/06/2019	B051	Bird	Carrion crow	10815	2167	11	479	2167-10815-11-479
112553	SAMEA112151052	B052-180619-01	18/06/2019	B052	Bird	Western jackdaw	7815	2168	11	2352	2168-7815-11-2352
112317	SAMEA112151053	B053-180619-01	18/06/2019	B053	Bird	Western jackdaw	10813	2627	11	2351	2627-10813-11-2351
112554	SAMEA112151054	B054-180619-01	18/06/2019	B054	Bird	Carrion crow	3536	2169	104	391	2169-3536-104-391
112555	SAMEA112151055	B054-180619-02	18/06/2019	B054	Bird	Carrion crow	801	2598	36	127	2598-801-36-127
111041	SAMEA112151056	B063-020719-01	02/07/2019	B063	Bird	Common swift	11384	2170	31	236	2170-11384-31-236
112556	SAMEA112151057	B063-020719-02	02/07/2019	B063	Bird	Common swift	22	277	8	70	277-22-8-70
112557	SAMEA112151058	B067-020719-01	02/07/2019	B067	Bird	Eurasian magpie	383	251	1	73	251-383-1-73
112558	SAMEA112151059	B068-020719-02	02/07/2019	B068	Bird	Eurasian magpie	383	251	1	73	251-383-1-73
112559	SAMEA112151060	B070-020719-01	02/07/2019	B070	Bird	Common blackbird	45	2171	7	44	2171-45-7-44
112560	SAMEA112151061	B071-020719-01	02/07/2019	B071	Bird	Carrion crow	45	2171	7	44	2171-45-7-44
112561	SAMEA112151062	B072-020719-02	02/07/2019	B072	Bird	Carrion crow	45	2171	7	44	2171-45-7-44
112562	SAMEA112151063	B073-020719-02	02/07/2019	B073	Bird	Carrion crow	3536	2169	104	391	2169-3536-104-391
112563	SAMEA112151064	B083-160719-02	16/07/2019	B083	Bird	Falcon	383	251	1	73	251-383-1-73
112564	SAMEA112151065	B086-160719-01	16/07/2019	B086	Bird	Pigeon	383	251	1	73	251-383-1-73
112565	SAMEA112151066	B096-300719-02	30/07/2019	B096	Bird	Carrion crow	951	2277	31	2358	2277-951-31-2358
112566	SAMEA112151067	B097-300719-02	30/07/2019	B097	Bird	Western jackdaw	801	2598	36	127	2598-801-36-127
112567	SAMEA112151068	B100-300719-01	30/07/2019	B100	Bird	Common swift	383	251	1	73	251-383-1-73
112568	SAMEA112151069	B100-300719-02	30/07/2019	B100	Bird	Common swift	475	827	5	67	827-475-5-67
112569	SAMEA112151070	B102-300719-02	30/07/2019	B102	Bird	Common blackbird	583	459	1	73	459-583-1-73
112570	SAMEA112151071	B103-300719-02	30/07/2019	B103	Bird	Common blackbird	177	2172	31	236	2172-177-31-236
112571	SAMEA112151072	B104-300719-01	30/07/2019	B104	Bird	Common blackbird	177	2172	31	236	2172-177-31-236
112572	SAMEA112151073	B108-130819-01	13/08/2019	B108	Bird	Falcon	19	2165	8	327	2165-19-8-327
112573	SAMEA112151074	B112-130819-01	13/08/2019	B112	Bird	Carrion crow	7882	2599	36	2357	2599-7882-36-2357
112574	SAMEA112151075	B113-130819-01	13/08/2019	B113	Bird	Carrion crow	801	2598	36	127	2598-801-36-127
112575	SAMEA112151076	B113-130819-02	13/08/2019	B113	Bird	Carrion crow	7836	2303	99	2347	2303-7836-99-2347
112576	SAMEA112151077	B114-130819-01	13/08/2019	B114	Bird	Common blackbird	10810	2173	101	2355	2173-10810-101-2355
112577	SAMEA112151078	B114-130819-03	13/08/2019	B114	Bird	Common blackbird	1277	2600	105	2359	2600-1277-105-2359
112578	SAMEA112151079	B119-270819-01	27/08/2019	B119	Bird	Eurasian magpie	11004	2601	106	1772	2601-11004-106-1772
112579	SAMEA112151080	B121-270819-02	27/08/2019	B121	Bird	Carrion crow	6591	2602	11	1634	2602-6591-11-1634

112580	SAMEA112151081	B122-270819-02	27/08/2019	B122	Bird	Common buzzard	19	2165	8	327	2165-19-8-327
112581	SAMEA112151082	B123-270819-01	27/08/2019	B123	Bird	Falcon	19	2165	8	327	2165-19-8-327
112583	SAMEA112151083	B158-221019-01	22/10/2019	B158	Bird	Carrion crow	5846	2304	40	858	2304-5846-40-858
112584	SAMEA112151084	B167-230720-01	23/07/2020	B167	Bird	Common swift	1044	84	27	25	84-1044-27-25
112585	SAMEA112151085	B183-300720-01	30/07/2020	B183	Bird	Common blackbird	11	414	7	217	414-11-7-217
112586	SAMEA112151086	B185-300720-01	30/07/2020	B185	Bird	Common blackbird	11	414	7	217	414-11-7-217
112587	SAMEA112151087	B189-130820-01	13/08/2020	B189	Bird	Common blackbird	8614	2612	36	6	2612-8614-36-6
112588	SAMEA112151088	B190-130820-01	13/08/2020	B190	Bird	Common blackbird	11	414	7	217	414-11-7-217
112590	SAMEA112151089	B193-200820-02	20/08/2020	B193	Bird	Duck	5207	2614	51	2356	2614-5207-51-2356
112591	SAMEA112151090	B194-200820-01	20/08/2020	B194	Bird	Egyptian goose	8614	2612	36	6	2612-8614-36-6
112592	SAMEA112151091	B198-200820-01	20/08/2020	B198	Bird	Egyptian goose	11	414	7	217	414-11-7-217
111042	SAMEA112151092	B203-270820-01	27/08/2020	B203	Bird	Carrion crow	11385	3163	80	203	3163-11385-80-203
111038	SAMEA112151093	B203-270820-02	27/08/2020	B203	Bird	Carrion crow	11381	3164	109	2368	3164-11381-109-2368
112593	SAMEA112151094	B203-270820-04	27/08/2020	B203	Bird	Carrion crow	677	36	19	276	36-677-19-276
112594	SAMEA112151095	B208-100920-01	10/09/2020	B208	Bird	Western jackdaw	677	36	19	276	36-677-19-276
112595	SAMEA112151096	B221-240920-01	24/09/2020	B221	Bird	Egyptian goose	1294	2460	13	152	2460-1294-13-152
111033	SAMEA112151097	B250-060321-01	06/03/2021	B250	Bird	Yellowhammer	11376	3165	108	2370	3165-11376-108-2370
112596	SAMEA112151098	B251-060321-01	06/03/2021	B251	Bird	Yellowhammer	11376	3165	108	2370	3165-11376-108-2370
112597	SAMEA112151099	B266-270321-01	27/03/2021	B266	Bird	Eurasian blackcap	6313	3166	107	2369	3166-6313-107-2369
112598	SAMEA112151100	B284-140421-02	14/04/2021	B284	Bird	Eurasian blackcap	11413	3257	22	2371	3257-11413-22-2371
112599	SAMEA112151101	B285-140421-02	14/04/2021	B285	Bird	Eurasian blackcap	1316	3167	74	463	3167-1316-74-463
112600	SAMEA112151102	B288-140421-02	14/04/2021	B288	Bird	Eurasian blackcap	6313	3168	107	2369	3168-6313-107-2369
112601	SAMEA112151103	B299-140421-01	14/04/2021	B299	Bird	Eurasian blackcap	6313	3168	107	2369	3168-6313-107-2369
112602	SAMEA112151104	B300-140421-01	14/04/2021	B300	Bird	Eurasian blackcap	1080	3169	74	130	3169-1080-74-130
112603	SAMEA112151105	B302-140421-01	14/04/2021	B302	Bird	Eurasian blackcap	6313	3168	107	2369	3168-6313-107-2369
112604	SAMEA112151106	B303-140421-01	14/04/2021	B303	Bird	Yellowhammer	11376	3165	108	2370	3165-11376-108-2370
112605	SAMEA112151107	B308-140421-01	14/04/2021	B308	Bird	Yellowhammer	11376	3165	108	2370	3165-11376-108-2370
112606	SAMEA112151108	B318-050521-01	05/05/2021	B318	Bird	Long-eared owl	45	2042	7	49	2042-45-7-49
112607	SAMEA112151109	B320-050521-02	05/05/2021	B320	Bird	Eurasian magpie	4570	3170	37	1795	3170-4570-37-1795
111040	SAMEA112151110	B321-050521-01	05/05/2021	B321	Bird	Eurasian magpie	11383	3171	11	1598	3171-11383-11-1598

112608	SAMEA112151111	B325-250521-01	25/05/2021	B325	Bird	Western jackdaw	563	3172	31	909	3172-563-31-909
112609	SAMEA112151112	B326-250521-01	25/05/2021	B326	Bird	Western jackdaw	4570	3170	37	1795	3170-4570-37-1795
111039	SAMEA112151113	B327-250521-01	25/05/2021	B327	Bird	Carrion crow	11382	3173	31	1468	3173-11382-31-1468
112610	SAMEA112151114	B328-250521-02	25/05/2021	B328	Bird	Carrion crow	6386	3174	36	406	3174-6386-36-406
112611	SAMEA112151115	B329-250521-01	25/05/2021	B329	Bird	Common blackbird	677	36	19	276	36-677-19-276
112612	SAMEA112151116	B330-250521-01	25/05/2021	B330	Bird	Starling	677	2070	19	276	2070-677-19-276
112613	SAMEA112151117	B331-250521-01	25/05/2021	B331	Bird	Great tit	45	297	7	49	297-45-7-49
111034	SAMEA112151118	B333-250521-01	25/05/2021	B333	Bird	Common blackbird	11377	3175	101	970	3175-11377-101-970
111298	SAMEA112151119	B335-070621-01	07/06/2021	B335	Bird	Western jackdaw	11584	3258	11	2372	3258-11584-11-2372
112614	SAMEA112151120	B336-070621-01	07/06/2021	B336	Bird	Western jackdaw	11379	3176	11	2367	3176-11379-11-2367
112615	SAMEA112151121	B336-070621-02	07/06/2021	B336	Bird	Western jackdaw	1481	3177	31	1836	3177-1481-31-1836
112944	SAMEA112151122	B337-070621-01	07/06/2021	B337	Bird	Eurasian magpie	1044	84	27	25	84-1044-27-25
111036	SAMEA112151123	B338-070621-02	07/06/2021	B338	Bird	Western jackdaw	11379	3178	11	2367	3178-11379-11-2367
112616	SAMEA112151124	B339-070621-01	07/06/2021	B339	Bird	Carrion crow	267	1407	1	73	1407-267-1-73
112617	SAMEA112151125	B342-070621-01	07/06/2021	B342	Bird	Great spotted woodpecker	19	82	8	7	82-19-8-7
112618	SAMEA112151126	B345-080621-01	08/06/2021	B345	Bird	Dunnock	383	251	1	73	251-383-1-73
112619	SAMEA112151127	B353-210621-01	21/06/2021	B353	Bird	Western jackdaw	448	2506	31	639	2506-448-31-639
112620	SAMEA112151128	B354-210621-01	21/06/2021	B354	Bird	Eurasian jay	2162	3179	7	634	3179-2162-7-634
112621	SAMEA112151129	B355-210621-01	21/06/2021	B355	Bird	Common chaffinch	267	2744	1	73	2744-267-1-73
111035	SAMEA112151130	B357-210621-01	21/06/2021	B357	Bird	Western jackdaw	11378	3180	7	1621	3180-11378-7-1621
112622	SAMEA112151131	B358-210621-01	21/06/2021	B358	Bird	Common blackbird	11	3181	7	217	3181-11-7-217
112623	SAMEA112151132	B361-210621-01	21/06/2021	B361	Bird	Common blackbird	230	2282	1	217	2282-230-1-217
112624	SAMEA112151133	B362-210621-01	21/06/2021	B362	Bird	Common blackbird	267	2744	1	73	2744-267-1-73
111037	SAMEA112151134	B364-210621-01	21/06/2021	B364	Bird	Eurasian magpie	11380	3182	11	200	3182-11380-11-200
112625	SAMEA112151135	W021-240919-01	24/09/2019	W021	Surface water	Moselle	704	2604	13	886	2604-704-13-886
112636	SAMEA112151136	W023-021219-02	02/12/2019	W023	Surface water	Alzette	538	1392	7	73	1392-538-7-73
112638	SAMEA112151137	W025-021219-02	02/12/2019	W025	Surface water	Alzette	11466	2278	100	2349	2278-11466-100-2349
112643	SAMEA112151138	W028-300120-01	30/01/2020	W028	Surface water	Alzette	9897	2279	9	18	2279-9897-9-18
112648	SAMEA112151139	W030-300120-02	30/01/2020	W030	Surface water	Alzette	9897	2279	9	18	2279-9897-9-18
112653	SAMEA112151140	W033-270220-02	27/02/2020	W033	Surface water	Alzette	4279	2280	31	1638	2280-4279-31-1638

111032	SAMEA112151141	W045-270820-05	27/08/2020	W045	Surface water	Alzette	11375	3183	10	92	3183-11375-10-92
112673	SAMEA112151142	W052-240920-02	24/09/2020	W052	Surface water	Sûre	991	2620	4	6	2620-991-4-6
112945	SAMEA112168221	W082-250521-04	25/05/2021	W082	Surface water	Alzette	45	663	7	49	663-45-7-49
112745	SAMEA112151143	W087-210621-01	21/06/2021	W087	Surface water	Alzette	10102	3184	11	2366	3184-10102-11-2366

Supplementary Table 2 – Metadata of human, animals and food *C. jejuni* isolates: PubMLST and ENA idea, strain ID, Sampling year, Sampling type, Sequence Type, Complex Type, allele of *gyrA* and *porA* and genotype.

PubMLST ID	ENA Biosample	Strain ID	Sampling year	Sampling type	ST	Complex Type	fn_gyrA	porA	Genotype
	SAMEA112765435	180430	2018	Chicken	50	53	1	6	53-50-1-6
	SAMEA112765436	180663	2018	Chicken	6175	543	9	1625	543-6175-9-1625
	SAMEA112765437	180667	2018	Chicken	257	72	10	1	72-257-10-1
	SAMEA112765538	190569	2019	Chicken	353	166	9	164	166-353-9-164
	SAMEA112765539	191062	2019	Poultry	400	219	15	98	219-400-15-98
	SAMEA112765580	200148	2020	Chicken	50	53	1	6	53-50-1-6
	SAMEA112765581	200220	2020	Chicken	6175	543	9	1625	543-6175-9-1625
114911	SAMEA112763457	200227	2020	Chicken	475	827	5	67	827-475-5-67
114912	SAMEA112763458	200776	2020	Turkey	19	82	8	7	82-19-8-7
	SAMEA112765584	200782	2020	Chicken	257	72	9	1	72-257-9-1
	SAMEA112765585	200869	2020	Chicken	257	72	10	1	72-257-10-1
114913	SAMEA112763459	200875	2020	Turkey	475	827	5	67	827-475-5-67
	SAMEA112765586	201032	2020	Chicken	122	377	3	7	377-122-3-7
	SAMEA112765654	210595	2021	Turkey	19	1355	8	1	1355-19-8-1
	SAMEA112765438	1800236	2018	Bovine	19	82	8	7	82-19-8-7
114914	SAMEA112763460	1800242	2018	Bovine	19	82	8	7	82-19-8-7
	SAMEA112765439	1803931	2018	Bovine	353	166	9	164	166-353-9-164
114915	SAMEA112763461	1807713	2018	Bovine	19	82	8	7	82-19-8-7
	SAMEA112765440	1807761	2018	Bovine	19	82	8	2023	82-19-8-2023
114916	SAMEA112763462	1900536	2019	Bovine	475	827	5	67	827-475-5-67
114917	SAMEA112763463	1903771	2019	Bovine	19	82	8	7	82-19-8-7
	SAMEA112765540	1905326	2019	Food	353	166	9	164	166-353-9-164
114918	SAMEA112763464	1908964	2019	Bovine	19	82	8	7	82-19-8-7
114919	SAMEA112763465	2001486	2020	Bovine	19	82	1	7	82-19-1-7
114920	SAMEA112763466	2001937	2020	Bovine	19	82	1	7	82-19-1-7
114921	SAMEA112763467	2004663	2020	Bovine	19	82	8	7	82-19-8-7
	SAMEA112765649	2101656	2021	Bovine	7355	2151	9	2360	2151-7355-9-2360
	SAMEA112765650	2103383	2021	Bovine	19	82	8	7	82-19-8-7
114923	SAMEA112763469	2103683	2021	Bovine	19	82	8	7	82-19-8-7

114924	SAMEA112763470	2104091	2021	Bovine	19	82	8	7	82-19-8-7
	SAMEA112765652	2104711	2021	Bovine	19	1355	8	1	1355-19-8-1
114945	SAMEA112763492	1802145-1	2018	Bovine	19	82	1	7	82-19-1-7
	SAMEA112765441	1809013-5	2018	Poultry	6175	543	9	1625	543-6175-9-1625
	SAMEA112765647	2009269-1	2020	Poultry	7355	2151	9	2360	2151-7355-9-2360
	SAMEA112765648	2009278-2	2020	Bovine	7355	2151	9	2360	2151-7355-9-2360
	SAMEA112765655	2106522-3	2021	Bovine	257	72	10	1	72-257-10-1
109854	SAMEA7489960	Camp036	2018	Bovine	19	82	8	7	82-19-8-7
109856	SAMEA7489961	Camp037	2018	Bovine	19	82	8	7	82-19-8-7
109881	SAMEA7489975	Camp052	2018	Human	2254	51	9	1	51-2254-9-1
109882	SAMEA7489976	Camp053	2018	Human	2254	51	9	1	51-2254-9-1
109931	SAMEA7489998	Camp086	2018	Chicken	6175	543	9	1625	543-6175-9-1625
109932	SAMEA7489999	Camp087	2018	Human	6175	543	9	1625	543-6175-9-1625
110226	SAMEA7490000	Camp088	2018	Human	6175	543	9	1625	543-6175-9-1625
109937	SAMEA7490001	Camp089	2018	Human	6175	543	9	1625	543-6175-9-1625
	SAMEA112765583	Camp121	2020	Chicken	19	1355	8	1	1355-19-8-1
	SAMEA112765641	Camp122	2020	Human	19	1355	8	1	1355-19-8-1
	SAMEA112765631	Camp123	2020	Human	50	1377	3	6	1377-50-3-6
	SAMEA112765645	Camp132	2020	Human	50	441	3	6	441-50-3-6
	SAMEA112765582	Camp137	2020	Chicken	21	50	1	7	50-21-1-7
114922	SAMEA112763468	Camp140	2020	Bovine	19	82	8	7	82-19-8-7
	SAMEA112765541	LNS0026606	2019	Human	6175	543	9	1625	543-6175-9-1625
	SAMEA112765664	LNS0034869	2021	Human	122	377	3	7	377-122-3-7
	SAMEA112765542	LNS0036110	2019	Human	257	72	10	1	72-257-10-1
	SAMEA112765604	LNS0037273	2020	Human	7355	2151	9	2360	2151-7355-9-2360
	SAMEA112765672	LNS0062340	2021	Human	21	50	1	7	50-21-1-7
	SAMEA112765442	LNS0075173	2018	Human	6175	543	9	1625	543-6175-9-1625
	SAMEA112765443	LNS0076217	2018	Human	6175	543	9	1625	543-6175-9-1625
	SAMEA112765679	LNS0130509	2021	Human	2254	51	9	1	51-2254-9-1
	SAMEA112765444	LNS0135444	2018	Human	50	1377	3	6	1377-50-3-6
	SAMEA112765445	LNS0166826	2018	Human	6175	543	9	1625	543-6175-9-1625
	SAMEA112765446	LNS0184308	2018	Human	50	441	3	6	441-50-3-6

	SAMEA112765447	LNS0213030	2018	Human	353	166	9	164	166-353-9-164
	SAMEA112765448	LNS0230559	2018	Human	6175	543	9	1625	543-6175-9-1625
	SAMEA112765449	LNS0277685	2018	Human	400	219	15	98	219-400-15-98
	SAMEA112765656	LNS0298000	2021	Human	50	441	3	6	441-50-3-6
	SAMEA112765543	LNS0333101	2019	Human	6175	543	9	1625	543-6175-9-1625
	SAMEA112765544	LNS0391085	2019	Human	50	53	1	6	53-50-1-6
	SAMEA112765450	LNS0432769	2018	Human	6175	543	9	1625	543-6175-9-1625
	SAMEA112765451	LNS0456874	2018	Human	50	441	3	6	441-50-3-6
	SAMEA112765640	LNS0458248	2020	Human	2254	51	9	1	51-2254-9-1
	SAMEA112765452	LNS0483513	2018	Human	400	219	15	98	219-400-15-98
	SAMEA112765453	LNS0620851	2018	Human	21	50	1	7	50-21-1-7
	SAMEA112765454	LNS0643347	2018	Human	2254	51	9	1	51-2254-9-1
	SAMEA112765665	LNS0666021	2021	Human	7355	2151	9	2360	2151-7355-9-2360
	SAMEA112765590	LNS0672454	2020	Human	122	377	3	7	377-122-3-7
	SAMEA112765624	LNS0692858	2020	Human	122	377	3	7	377-122-3-7
	SAMEA112765455	LNS0721049	2018	Human	353	166	9	164	166-353-9-164
	SAMEA112765625	LNS0766413	2020	Human	7355	2151	9	2360	2151-7355-9-2360
	SAMEA112765545	LNS0799124	2019	Human	400	219	15	98	219-400-15-98
	SAMEA112765659	LNS0841228	2021	Human	19	1355	8	1	1355-19-8-1
	SAMEA112765456	LNS0899902	2018	Human	50	53	1	6	53-50-1-6
	SAMEA112765612	LNS0926723	2020	Human	19	1355	8	1	1355-19-8-1
	SAMEA112765546	LNS0927051	2019	Human	353	166	9	164	166-353-9-164
	SAMEA112765618	LNS0948878	2020	Human	7355	2151	9	2360	2151-7355-9-2360
114928	SAMEA112763474	LNS1096056	2018	Human	19	82	8	7	82-19-8-7
	SAMEA112765457	LNS1186273	2018	Human	6175	543	9	1625	543-6175-9-1625
	SAMEA112765547	LNS1249920	2019	Human	21	50	1	7	50-21-1-7
	SAMEA112765587	LNS1271330	2020	Human	50	441	3	6	441-50-3-6
	SAMEA112765595	LNS1278551	2020	Human	6175	543	9	1625	543-6175-9-1625
	SAMEA112765458	LNS1282200	2018	Human	400	219	15	98	219-400-15-98
	SAMEA112765606	LNS1288690	2020	Human	257	72	10	1	72-257-10-1
	SAMEA112765548	LNS1304848	2019	Human	6175	543	9	1625	543-6175-9-1625
	SAMEA112765549	LNS1380494	2019	Human	21	50	1	7	50-21-1-7

	SAMEA112765600	LNS1410767	2020	Human	2254	51	9	1	51-2254-9-1
	SAMEA112765610	LNS1467429	2020	Human	10045	2063	3	60	2063-10045-3-60
	SAMEA112765671	LNS1470215	2021	Human	10846	2379	8	2	2379-10846-8-2
	SAMEA112765459	LNS1515213	2018	Human	6175	543	9	1625	543-6175-9-1625
	SAMEA112765460	LNS1574307	2018	Human	6175	543	9	1625	543-6175-9-1625
	SAMEA112765461	LNS1578743	2018	Human	353	166	9	164	166-353-9-164
	SAMEA112765462	LNS1662782	2018	Human	50	53	1	6	53-50-1-6
	SAMEA112765591	LNS1668617	2020	Human	7355	2151	9	2360	2151-7355-9-2360
	SAMEA112765463	LNS1668621	2018	Human	6175	543	9	1625	543-6175-9-1625
	SAMEA112765464	LNS1675319	2018	Human	6175	543	9	1625	543-6175-9-1625
	SAMEA112765550	LNS1725997	2019	Human	10045	2063	3	60	2063-10045-3-60
	SAMEA112765465	LNS1754341	2018	Human	19	1355	8	1	1355-19-8-1
	SAMEA112765632	LNS1806192	2020	Human	122	377	3	7	377-122-3-7
	SAMEA112765676	LNS1821686	2021	Human	50	53	1	6	53-50-1-6
114943	SAMEA112763490	LNS1834063	2021	Human	19	82	8	7	82-19-8-7
110015	SAMEA112763484	LNS1866633	2019	Human	19	82	8	7	82-19-8-7
	SAMEA112765551	LNS1881298	2019	Human	50	53	1	6	53-50-1-6
	SAMEA112765628	LNS1958743	2020	Human	122	377	3	7	377-122-3-7
114933	SAMEA112763479	LNS1987825	2018	Human	475	827	5	67	827-475-5-67
114938	SAMEA112763485	LNS2069990	2019	Human	475	827	5	67	827-475-5-67
114930	SAMEA112763476	LNS2124172	2018	Human	19	82	8	7	82-19-8-7
	SAMEA112765466	LNS2136373	2018	Human	50	441	3	6	441-50-3-6
	SAMEA112765592	LNS2163380	2020	Human	10045	2063	3	60	2063-10045-3-60
	SAMEA112765668	LNS2167432	2021	Human	10846	2379	8	2	2379-10846-8-2
	SAMEA112765467	LNS2266426	2018	Human	353	166	9	164	166-353-9-164
	SAMEA112765468	LNS2299557	2018	Human	6175	543	9	1625	543-6175-9-1625
	SAMEA112765469	LNS2335828	2018	Human	353	166	9	164	166-353-9-164
	SAMEA112765607	LNS2407788	2020	Human	2254	51	9	1	51-2254-9-1
	SAMEA112765552	LNS2436514	2019	Human	6175	543	9	1625	543-6175-9-1625
	SAMEA112765633	LNS2460455	2020	Human	257	72	10	1	72-257-10-1
	SAMEA112765553	LNS2468064	2019	Human	6175	543	9	1625	543-6175-9-1625
	SAMEA112765639	LNS2469830	2020	Human	10846	2379	8	2	2379-10846-8-2

114934	SAMEA112763480	LNS2470460	2018	Human	475	827	5	67	827-475-5-67
	SAMEA112765658	LNS2506582	2021	Human	257	72	10	1	72-257-10-1
	SAMEA112765554	LNS2641285	2019	Human	10045	2063	3	60	2063-10045-3-60
114931	SAMEA112763477	LNS2671467	2018	Human	19	82	8	7	82-19-8-7
	SAMEA112765619	LNS2680632	2020	Human	50	1377	3	6	1377-50-3-6
114932	SAMEA112763478	LNS2772674	2018	Human	19	82	8	7	82-19-8-7
114935	SAMEA112763481	LNS2986469	2019	Human	19	82	8	7	82-19-8-7
	SAMEA112765616	LNS3025831	2020	Human	7355	2151	9	2360	2151-7355-9-2360
	SAMEA112765470	LNS3090015	2018	Human	50	441	3	6	441-50-3-6
	SAMEA112765471	LNS3149868	2018	Human	6175	543	9	1625	543-6175-9-1625
	SAMEA112765611	LNS3172954	2020	Human	7355	2151	9	2360	2151-7355-9-2360
	SAMEA112765472	LNS3220880	2018	Human	6175	543	9	1625	543-6175-9-1625
	SAMEA112765653	LNS3245052	2021	Human	10846	2379	8	2	2379-10846-8-2
114927	SAMEA112763473	LNS3267197	2018	Human	1044	84	27	25	84-1044-27-25
114925	SAMEA112763471	LNS3278856	2018	Human	475	827	5	67	827-475-5-67
	SAMEA112765685	LNS3301082	2021	Human	6175	543	9	1625	543-6175-9-1625
	SAMEA112765598	LNS3326294	2020	Human	6175	543	9	1625	543-6175-9-1625
	SAMEA112765683	LNS3429263	2021	Human	122	377	3	7	377-122-3-7
	SAMEA112765630	LNS3461998	2020	Human	122	377	3	7	377-122-3-7
	SAMEA112765473	LNS3498860	2018	Human	353	166	9	164	166-353-9-164
	SAMEA112765474	LNS3553881	2018	Human	353	166	9	164	166-353-9-164
	SAMEA112765555	LNS3603719	2019	Human	6175	543	9	1625	543-6175-9-1625
	SAMEA112765475	LNS3663438	2018	Human	400	219	15	98	219-400-15-98
	SAMEA112765476	LNS3669946	2018	Human	400	219	15	98	219-400-15-98
	SAMEA112765637	LNS3673907	2020	Human	7355	2151	9	2360	2151-7355-9-2360
	SAMEA112765556	LNS3780275	2019	Human	50	1377	3	6	1377-50-3-6
	SAMEA112765477	LNS3782382	2018	Human	6175	543	9	1625	543-6175-9-1625
	SAMEA112765478	LNS3811300	2018	Human	400	219	15	98	219-400-15-98
	SAMEA112765667	LNS3814770	2021	Human	21	50	1	7	50-21-1-7
	SAMEA112765617	LNS3826485	2020	Human	7355	2151	9	2360	2151-7355-9-2360
	SAMEA112765557	LNS3839297	2019	Human	400	219	15	98	219-400-15-98
	SAMEA112765479	LNS3849481	2018	Human	353	166	9	164	166-353-9-164

SAMEA112765480	LNS3851048	2018	Human	400	219	15	98	219-400-15-98
SAMEA112765481	LNS3878735	2018	Human	50	441	3	6	441-50-3-6
SAMEA112765601	LNS3909174	2020	Human	10045	2063	3	60	2063-10045-3-60
SAMEA112765558	LNS3923052	2019	Human	257	72	10	1	72-257-10-1
SAMEA112765673	LNS3971881	2021	Human	10846	2379	8	2	2379-10846-8-2
SAMEA112765482	LNS3996841	2018	Human	353	166	9	164	166-353-9-164
SAMEA112765483	LNS4032871	2018	Human	257	72	10	1	72-257-10-1
SAMEA112765484	LNS4078899	2018	Human	353	166	9	164	166-353-9-164
SAMEA112765485	LNS4086976	2018	Human	400	219	15	98	219-400-15-98
SAMEA112765642	LNS4127269	2020	Human	122	377	3	7	377-122-3-7
SAMEA112765638	LNS4257815	2020	Human	7355	2151	9	2360	2151-7355-9-2360
SAMEA112765675	LNS4326122	2021	Human	122	377	3	7	377-122-3-7
SAMEA112765559	LNS4335150	2019	Human	6175	543	9	1625	543-6175-9-1625
SAMEA112765635	LNS4348168	2020	Human	7355	2151	9	2360	2151-7355-9-2360
SAMEA112765602	LNS4385826	2020	Human	6175	543	9	1625	543-6175-9-1625
SAMEA112765560	LNS4495349	2019	Human	10045	2063	3	60	2063-10045-3-60
SAMEA112765486	LNS4511334	2018	Human	21	50	1	7	50-21-1-7
SAMEA112765487	LNS4571000	2018	Human	50	53	1	6	53-50-1-6
SAMEA112765599	LNS4633233	2020	Human	50	1377	3	6	1377-50-3-6
SAMEA112765629	LNS4654607	2020	Human	400	219	15	98	219-400-15-98
SAMEA112765643	LNS4663040	2020	Human	19	1355	8	1	1355-19-8-1
SAMEA112765684	LNS4674810	2021	Human	257	72	10	1	72-257-10-1
SAMEA112765488	LNS4704353	2018	Human	50	441	3	6	441-50-3-6
SAMEA112765489	LNS4760909	2018	Human	122	377	3	7	377-122-3-7
SAMEA112765669	LNS4766033	2021	Human	10846	2379	8	2	2379-10846-8-2
SAMEA112765609	LNS4778500	2020	Human	19	1355	8	1	1355-19-8-1
SAMEA112765490	LNS4786690	2018	Human	6175	543	9	1625	543-6175-9-1625
SAMEA112765561	LNS4808088	2019	Human	50	441	3	6	441-50-3-6
SAMEA112765674	LNS4817241	2021	Human	2254	51	9	1	51-2254-9-1
SAMEA112765491	LNS4885850	2018	Human	19	1355	8	1	1355-19-8-1
SAMEA112765636	LNS4891524	2020	Human	10846	2379	8	2	2379-10846-8-2
SAMEA112765492	LNS4917969	2018	Human	6175	543	9	1625	543-6175-9-1625

	SAMEA112765493	LNS4926602	2018	Human	6175	543	9	1625	543-6175-9-1625
	SAMEA112765562	LNS4926940	2019	Human	50	441	3	6	441-50-3-6
	SAMEA112765670	LNS4939672	2021	Human	21	50	1	7	50-21-1-7
	SAMEA112765603	LNS5004765	2020	Human	7355	2151	9	2360	2151-7355-9-2360
	SAMEA112765563	LNS5062104	2019	Human	6175	543	9	1625	543-6175-9-1625
	SAMEA112765597	LNS5149213	2020	Human	2254	51	9	1	51-2254-9-1
114944	SAMEA112763491	LNS5178364	2021	Human	1044	84	27	25	84-1044-27-25
	SAMEA112765494	LNS5184657	2018	Human	50	441	3	6	441-50-3-6
	SAMEA112765614	LNS5239796	2020	Human	19	1355	8	1	1355-19-8-1
114937	SAMEA112763483	LNS5328171	2019	Human	19	82	8	7	82-19-8-7
114940	SAMEA112763487	LNS5390735	2020	Human	1044	84	27	25	84-1044-27-25
114941	SAMEA112763488	LNS5392364	2021	Human	19	82	8	7	82-19-8-7
	SAMEA112765564	LNS5409610	2019	Human	257	72	10	1	72-257-10-1
	SAMEA112765495	LNS5480964	2018	Human	6175	543	9	1625	543-6175-9-1625
	SAMEA112765496	LNS5554270	2018	Human	353	166	9	164	166-353-9-164
	SAMEA112765497	LNS5556180	2018	Human	6175	543	9	1625	543-6175-9-1625
	SAMEA112765498	LNS5565670	2018	Human	353	166	9	164	166-353-9-164
	SAMEA112765499	LNS5625996	2018	Human	2254	51	9	1	51-2254-9-1
	SAMEA112765662	LNS5684448	2021	Human	10846	2379	8	2	2379-10846-8-2
	SAMEA112765500	LNS5700864	2018	Human	6175	543	9	1625	543-6175-9-1625
	SAMEA112765661	LNS5730504	2021	Human	21	50	1	7	50-21-1-7
	SAMEA112765644	LNS5733036	2020	Human	10846	2379	8	2	2379-10846-8-2
114929	SAMEA112763475	LNS5803147	2018	Human	475	827	3	67	827-475-3-67
	SAMEA112765605	LNS5965469	2020	Human	6175	543	9	1625	543-6175-9-1625
	SAMEA112765501	LNS5967632	2018	Human	122	377	3	7	377-122-3-7
	SAMEA112765678	LNS5971235	2021	Human	353	166	9	131	166-353-9-131
	SAMEA112765660	LNS5998684	2021	Human	2254	51	9	1	51-2254-9-1
	SAMEA112765565	LNS6042390	2019	Human	6175	543	9	1625	543-6175-9-1625
	SAMEA112765502	LNS6067339	2018	Humain	353	166	9	164	166-353-9-164
	SAMEA112765626	LNS6102005	2020	Human	6175	543	9	1625	543-6175-9-1625
	SAMEA112765503	LNS6102664	2018	Human	50	441	3	6	441-50-3-6
	SAMEA112765504	LNS6259234	2018	Human	50	441	3	6	441-50-3-6

	SAMEA112765613	LNS6315603	2020	Human	19	1355	8	1	1355-19-8-1
	SAMEA112765663	LNS6357384	2021	Human	10045	2063	3	60	2063-10045-3-60
	SAMEA112765505	LNS6409576	2018	Human	6175	543	9	1625	543-6175-9-1625
	SAMEA112765566	LNS6432252	2019	Human	50	53	1	6	53-50-1-6
	SAMEA112765506	LNS6447723	2018	Human	2254	51	9	1	51-2254-9-1
	SAMEA112765507	LNS6547049	2018	Human	6175	543	9	1625	543-6175-9-1625
	SAMEA112765508	LNS6557340	2018	Humain	353	166	9	164	166-353-9-164
	SAMEA112765596	LNS6574677	2020	Human	6175	543	9	1625	543-6175-9-1625
	SAMEA112765623	LNS6590045	2020	Human	7355	2151	9	2360	2151-7355-9-2360
	SAMEA112765567	LNS6665366	2019	Human	50	53	1	6	53-50-1-6
	SAMEA112765509	LNS6735307	2018	Human	6175	543	9	1625	543-6175-9-1625
	SAMEA112765680	LNS6784486	2021	Human	50	441	3	6	441-50-3-6
	SAMEA112765510	LNS6843297	2018	Human	6175	543	9	1625	543-6175-9-1625
	SAMEA112765568	LNS6945529	2019	Human	122	377	3	7	377-122-3-7
	SAMEA112765622	LNS6997530	2020	Human	50	1377	3	6	1377-50-3-6
	SAMEA112765511	LNS6999135	2018	Human	353	166	9	164	166-353-9-164
	SAMEA112765512	LNS7035520	2018	Human	2254	51	9	1	51-2254-9-1
	SAMEA112765594	LNS7051397	2020	Human	10045	2063	3	60	2063-10045-3-60
	SAMEA112765569	LNS7177981	2019	Human	50	1377	3	6	1377-50-3-6
	SAMEA112765620	LNS7206545	2020	Human	50	1377	3	6	1377-50-3-6
	SAMEA112765513	LNS7210357	2018	Human	400	219	15	98	219-400-15-98
	SAMEA112765646	LNS7265199	2020	Human	10846	2379	8	2	2379-10846-8-2
	SAMEA112765514	LNS7265268	2018	Human	50	441	3	6	441-50-3-6
	SAMEA112765570	LNS7298634	2019	Human	6175	543	9	1625	543-6175-9-1625
	SAMEA112765515	LNS7304798	2018	Human	400	219	15	98	219-400-15-98
114936	SAMEA112763482	LNS7314261	2019	Human	19	82	8	7	82-19-8-7
	SAMEA112765571	LNS7389331	2019	Human	122	377	3	7	377-122-3-7
	SAMEA112765516	LNS7463476	2018	Human	6175	543	9	1625	543-6175-9-1625
	SAMEA112765572	LNS7647010	2019	Human	6175	543	9	1625	543-6175-9-1625
	SAMEA112765517	LNS7653924	2018	Human	400	219	15	98	219-400-15-98
	SAMEA112765518	LNS7665344	2018	Human	400	219	15	98	219-400-15-98
	SAMEA112765519	LNS7684455	2018	Human	50	441	3	6	441-50-3-6

	SAMEA112765520	LNS7685200	2018	Human	50	441	3	6	441-50-3-6
	SAMEA112765573	LNS7718383	2019	Human	21	50	1	7	50-21-1-7
114942	SAMEA112763489	LNS7784891	2021	Human	19	82	8	7	82-19-8-7
	SAMEA112765589	LNS7841065	2020	Human	122	377	3	7	377-122-3-7
	SAMEA112765574	LNS7858885	2019	Human	122	377	3	7	377-122-3-7
	SAMEA112765666	LNS7859802	2021	Human	10846	2379	8	2	2379-10846-8-2
	SAMEA112765521	LNS7866515	2018	Human	6175	543	9	1625	543-6175-9-1625
	SAMEA112765522	LNS8050392	2018	Human	353	166	9	164	166-353-9-164
	SAMEA112765593	LNS8055349	2020	Human	10045	2063	3	60	2063-10045-3-60
	SAMEA112765523	LNS8091791	2018	Human	353	166	9	164	166-353-9-164
	SAMEA112765575	LNS8113426	2019	Human	50	1377	3	6	1377-50-3-6
	SAMEA112765524	LNS8138066	2018	Human	6175	543	9	1625	543-6175-9-1625
	SAMEA112765525	LNS8138670	2018	Human	6175	543	9	1625	543-6175-9-1625
	SAMEA112765576	LNS8170996	2019	Human	257	72	10	1	72-257-10-1
	SAMEA112765526	LNS8188016	2018	Human	353	166	9	164	166-353-9-164
	SAMEA112765527	LNS8401581	2018	Human	353	166	9	164	166-353-9-164
	SAMEA112765682	LNS8452384	2021	Human	6175	543	9	1625	543-6175-9-1625
	SAMEA112765634	LNS8508707	2020	Human	10846	2379	8	2	2379-10846-8-2
	SAMEA112765577	LNS8527914	2019	Human	10045	2063	3	60	2063-10045-3-60
	SAMEA112765657	LNS8562189	2021	Human	10846	2379	8	2	2379-10846-8-2
	SAMEA112765578	LNS8564581	2019	Human	400	219	15	98	219-400-15-98
	SAMEA112765588	LNS8688859	2020	Human	122	377	3	7	377-122-3-7
114939	SAMEA112763486	LNS8719332	2020	Human	1044	84	27	25	84-1044-27-25
	SAMEA112765681	LNS8729088	2021	Human	6175	543	9	1625	543-6175-9-1625
	SAMEA112765621	LNS8865000	2020	Human	50	1377	3	6	1377-50-3-6
	SAMEA112765677	LNS8907005	2021	Human	19	1355	8	1	1355-19-8-1
	SAMEA112765528	LNS9158783	2018	Human	50	1377	3	6	1377-50-3-6
	SAMEA112765529	LNS9184192	2018	Human	21	50	1	7	50-21-1-7
	SAMEA112765627	LNS9241924	2020	Human	10846	2379	8	2	2379-10846-8-2
	SAMEA112765530	LNS9262320	2018	Human	50	441	3	6	441-50-3-6
	SAMEA112765531	LNS9287348	2018	Human	400	219	15	98	219-400-15-98
	SAMEA112765579	LNS9388969	2019	Human	21	50	1	7	50-21-1-7

	SAMEA112765532	LNS9444847	2018	Human	353	166	9	164	166-353-9-164
	SAMEA112765608	LNS9473484	2020	Human	7355	2151	9	2360	2151-7355-9-2360
	SAMEA112765533	LNS9478489	2018	Human	50	441	3	6	441-50-3-6
	SAMEA112765534	LNS9515553	2018	Human	353	166	9	164	166-353-9-164
	SAMEA112765535	LNS9539548	2018	Human	6175	543	9	1625	543-6175-9-1625
	SAMEA112765536	LNS9665428	2018	Human	353	166	9	164	166-353-9-164
	SAMEA112765651	LNS9725252	2021	Human	10846	2379	8	2	2379-10846-8-2
114926	SAMEA112763472	LNS9806031	2018	Human	1044	84	27	25	84-1044-27-25
	SAMEA112765615	LNS9833853	2020	Human	257	72	10	1	72-257-10-1
	SAMEA112765537	LNS9959957	2018	Human	50	53	1	6	53-50-1-6

Supplementary Table 3 - Number of human recurring genotypes isolated between 2018 and 2021 and number of these isolates found in food and animal samples. The largest number of human recurring genotypes based per one-year periods is highlighted in bold. The letter corresponds to the origin of food and animal isolates: b, bovine; c, chicken; f, food with species not defined; p; poultry; t, turkey.

	**Number of human isolates**					**Food and animal isolates**			
**Recurring genotypes**	**2018**	**2019**	**2020**	**2021**	**Total**	**2018**	**2019**	**2020**	**2021**
543-6175-9-1625	**32**	**11**	6	3	**52**	2(c), 1(p)			1(c)
166-353-9-164	22	1		1	24	1(b)	1(c), 1(f)		
441-50-3-6	14	2	2	2	20				
219-400-15-98	13	3	1		17		1(p)		
377-122-3-7	2	3	8	3	16			1(c)	
2379-10846-8-2			6	**9**	15				
2151-7355-9-2360			**13**	1	14			1(b), 1(p)	1(b)
51-2254-9-1	6		4	3	13				
1377-50-3-6	2	3	6		11			1(c)	
50-21-1-7	3	4		4	11			1(c)	
82-19-8-7	4	4		3	11	5(b)	2(b)	4(b), 1(t)	3(b)
1355-19-8-1	2		6	2	10			1(c)	1(b), 1(t)
2063-10045-3-60		4	5	1	10				
72-257-10-1	1	4	3	2	10	1(c)		2(c)	1(b)
53A-50-1-6	4	4		1	9	1(c)		1(c)	
17-21-1-48	5	3	1		9	2(b)		2(b)	1(b)
277-22-1-70	5	1	1	2	9	1(b), 1(f)			
1837-2079-3-67	5	3			8				
46B-21-8-7	4	1	1	2	8	5(b)	3(b)	2(b)	2(b)
75-464-8-1678	7	1			8				
147-44-8-11	4	1	1	1	7		1(c)		
1600-50-8-6	2	1		4	7				
2149-10298-8-2364			6	1	7			1(c)	
662-8334-9-98	3	2	1	1	7				
85-5018-1-51	6	1			7				
68B-354-8-957	2	2	2		6			1(t)	
87-48-3-14	4		1	1	6	1(c)			
1385-8579-8-957	5				5				
1451-7517-10-10	4	1			5		1(p)		
153-48-5-7	3			2	5			1(c)	

1615-50-8-6	3	1	1		5			
1660-354-8-58	2	3			5			
1909-48-5-6		5			5			
2226-122-3-7			3	2	5			1(b)
2974-11328-5-14				5	5			
313-2274-9-705	4		1		5			
46A-21-8-2068	5				5			
827-475-5-67	4	1			5	1(b)	1(c), 1(t)	
84-1044-27-25	2		2	1	5			
914-441-9-115	3			2	5			
1333-19-1-327	3		1		4			1(c)
1461-21-8-37	4				4		1(b)	
1601-48-5-7	4				4			
1868-10025-3-92	1	3			4	1(c), 2(p)	3(c)	
2230-904-17-6			3	1	4		2(c)	
295-52-10-1	4				4			
54-257-10-360		1		3	4			
595A-3155-8-2059	4				4			
732A-53-1-27		4			4			
732B-53-1-331			4		4		1(c)	
1555-52-9-1	3				3			
2047-257-10-1		3			3			
2054-658-27-25		3			3			
2145-52-9-1			3		3			
288-21-1-7		3			3			
527-3155-8-2059	3				3			
53B-50-1-80		3			3			
595B-3155-93-2059	3				3			
68A-354-8-2207	3				3			

Supplementary Table 4 – Concordance between genotyping CT(SeqSphere)-ST-gyrA-porA and the BIGsdb genotyping and the number of isolates corresponding.

**Genotypes**	**Correspondence BIGsdb (AD)***	**Collection Luxembourg in BIGsdb**						**Collection BIGsdb**
		**No. of isolates**						
**CT-ST-*gyrA-porA***		Human	Wild Birds	Food	Ruminant	Poultry	Environment	Total No. of isolates
82-19-8-7	Cjc_cgc_10 group_**59** (10)	27	1	1	24	3	2	356
84-1044-27-25	Cjc_cgc_10 group_**1159** (10)	2	1				1	4
827-475-5-67	Cjc_cgc_25 group_**164** (25)	4	1	1	1	1		42

*AD = Allele Distance defined as cutoff
